# Protective Effect of *Artemisia annua* L. Extract against Galactose-Induced Oxidative Stress in Mice

**DOI:** 10.1371/journal.pone.0101486

**Published:** 2014-07-02

**Authors:** Mi Hye Kim, Ji Yeon Seo, Kwang Hyun Liu, Jong-Sang Kim

**Affiliations:** 1 School of Food Science and Biotechnology, Kyungpook National University, Daegu, Republic of Korea; 2 College of Pharmacy, Kyungpook National University, Daegu, Republic of Korea; Oregon Health & Science University, United States of America

## Abstract

*Artemisia annua* L. (also called qinghao) has been well known as a source of antimalarial drug artemisinins. In addition, the herb was reported to have in vitro antioxidative activity. The present study investigated the protective effect of aqueous ethanol extract of Qinghao (AA extract) against D-galactose-induced oxidative stress in C57BL/6J mice. Feeding AA extract-containing diet lowered serum levels of malondialdehyde and 8-OH-dG that are biomarkers for lipid peroxidation and DNA damage, respectively. Furthermore, AA extract feeding enhanced the activity of NQO1, a typical antioxidant marker enzyme, in tissues such as kidney, stomach, small intestine, and large intestine. In conclusion, AA extract was found to have antioxidative activity in mouse model.

## Introduction

*Artemisia annua* L., which is also known as ‘sweet wormwood’ and ‘Qinghao’, has traditionally been used in China for the treatment of fever and chills [1]. The herb has been reported to contain various bioactive compounds. In particular, artemisinin and its derivatives have been clinically used to treat drug-resistant malaria while they were reported to have several bioactive functions including antitumor and anti-inflammatory activities [2], [3]. In addition, coumarins, flavonoids, and other terpenoids constituents present in *A. annua* L. are also reported to have significant pharmacological activities such as antitumor and antibacterial activities that contribute to the therapeutic effects of the herb [4], [5]. The total antioxidant capacity (ORAC) of *A. annua* leaves extract was reported as 1,125 µmoles of Trolox equiv/g, which is half of the ORAC of oregano (the highest reported ORAC for an herb) extracts [6].

Some flavonoids and sesquiterpenes were well known to induce antioxidant and phase 2 detoxifying enzymes such as NAD(P)H:quinone oxidoreductase 1 (NQO1), heme oxygenase 1, γ-glutamylcycteine ligase, glutathione reductase and glutathione *S*-transferase [7]–[11] as well as have direct radical scavenging activity [12], [13]. Therefore, *A. annua* rich in flavonoids and sesquiterpenes is most like to have capability to induce antioxidant enzymes and exert in vivo antioxidative activity. Although in vitro antioxidant potential of *A. annua* L. extract was reported [14], the in vivo antioxidant activity of the herbal extract has not been studied so far to our best knowledge.

D-Galactose is normally metabolized by galactose-1-phosphate uridyltransferase and D-galactokinase. A large dose of D-galactose will be converted to galactitol and cause an osmotic stress, generating reactive oxygen species (ROS) [15], [16]. Therefore, we investigated the antioxidative effect of 80% (v/v) ethanol extract of *A. annua* in mice chronically exposed to D-galactose.

## Materials and Methods

### Materials

All reagents used were of ACS grade, and most of them were purchased from Sigma Aldrich (St. Louis, MO, USA). Dried aerial part of *A. annua* L. was provided from Goksung Heungsan Farmers Cooprerative Ltd. (Goksung, Chunnam, Republic of Korea). A voucher (No. 100) is deposited at Kyungppok National University in Daegu, Republic of Korea.

### Preparation of *A. annua* extract

Dried aerial part of *A. annua* L. harvested in June, 2012 was powdered using Cyclotec mill (Tecator, Sweden) and extracted with 20 volumes of 80%(v/v) ethanol for 12 h at 80°C, followed by concentration by rotary evaporation at 40°C and freeze drying to make powder (AA extract) [17].

### LC-MS/MS analysis of artemisinin

The concentration of artemisinin in *Artemisia annua* L. extracts was quantified using LC-MS/MS as follows; The system consisted of a Thermo Vantage triple-quadrupole mass spectrometer (Thermo Fisher Scientific, San Jose, CA), coupled with a Thermo Accela HPLC system (Thermo Fisher Scientific). Artemisinin was separated on a Luna phenyl-hexyl column (2×100 mm, 3 m, Phenomenex, Torrance, CA) with an isocratic mobile phase consisting of acetonitrile and water (70/30, v/v) containing 0.1% formic acid. The mobile phase was eluted at a flow rate of 0.2 mL/min. For quantification of artemisinin, mass spectra were recorded by electrospray ionization in positive mode. The operating conditions were optimized by flow injection of an analyte and were as follows: capillary temperature, 350°C; vaporizer temperature, 300°C; sheath gas pressure, 35 arbitrary units; auxiliary gas, 10 arbitrary units; nitrogen gas flow rate, 8 L/min; spray voltage, 4,000 V; collision energy, 30 eV. Quantitation was performed by selective reaction monitoring of the protonated precursor ion [M+H]+ and the related product ion for artemisinin. Ions were detected by monitoring the transitions of m/z 283→247 for artemisinin. The analytical data were processed by Xcalibur (version 2.1) software (Thermo Fisher Scientific). The lower limit of quantitation for the analyte was 10 mg/mL. The inter-assay precision for the analyte was less than 15%.

### Animal experiments

Male C57BL/6J mouse (5-week old) were obtained from Daehan Biolink (Eumsung, Chungbuk, Republic of Korea) and were housed in plastic cages at a constant temperature of 22±2°C and 50% humidity under a 12-h light/dark cycles. Animals were given freely a standard diet (Chow, Purina, Korea) and water.

After adaptation period of a week, mice were randomized by weight into eight groups (10 mice per group) and fed the diet prepared based on AIN-76A formulation throughout the experimental period. The eight dietary groups consist of; (i) negative control with no treatment (s.c. injected with 0.9% saline) (ii) vitamin E (150 IU/kg diet) alone, (iii) AA extract alone (50 g/kg diet), (iv) daily s.c. injection of D-galactose (100 mg/kg bw) alone, daily s.c. injection of D-galactose plus dietary vitamin E (150 IU/kg diet) (v), or AA extract 10 (vi), 20 (vii), 50 g/kg(viii) for 6 weeks before sacrifice. More specifically, mice in positive control group (group iv) were injected subcutaneously with D-galactose (100 mg/kg bw) dissolved in 0.9% saline solution every day until sacrifice. Animals in experimental groups were allowed free access to diets containing different levels of AA extract. Control and experimental groups of mice were euthanized by CO_2_ inhalation after treatment, at which time livers, kidney, stomach, small intestine, large intestine were collected, weighed, and subjected to biochemical assays, histologic examination.

### Ethics Statement

All animal care and handling including surgical procedures was approved by the Animal Ethics Committee of Kyungpook National University (IRB permission number: KNU 2013-64). All efforts were made to minimize suffering. Care administered to the animals was in accordance with guidelines for the Care and Use of Laboratory Animals of the National Institutes of Health.

### Preparation of tissue homogenates and blood collection

Organs such as liver, stomach, kidney, lung, small intestine mucosa, large intestine mucosa were collected right after the CO_2_ asphyxiation of mice. Each tissue (100 mg) was homogenized with 0.4 mL of phosphate buffered saline (PBS) or Triton X lysis buffer (pH 7.4) containing protease inhibitor. Tissue extracts were centrifuged at 12,000×g for 1 h and supernatant was collected and stored at −70°C until further analysis. Blood was collected from infraorbital vein, followed by centrifugation at 3,000×g for 20 min to separate serum and stored at −20°C. Every experiment was conducted on ice.

### Antioxidative activity assay for *A. annua* extract

Oxidative stress was quantified in cells by 2,7-dichlorofluorescein (DCF) assay according to Wang and Josep[18], with slight modifications. A human hepatoma cell line (HepG2 cells) was obtained from ATCC (Manassas, VA, USA). For routine maintenance, cells were grown in α-MEM (Gibco, Grand Island, NY) supplemented with 10% heat-inactivated fetal bovine serum (FBS) at 37°C in an atmosphere of 5% CO_2_/ 5% air under saturating humidity and passaged every other day (1∶4 split ratio) by trypsinization with 0.25% trypsin/0.02% EDTA sodium salt solution (Thermo Fisher Scientific Inc., Waltham, MA, USA).

The cells (2,000 cells per well) were seeded into black-bottom 96-well plate, cultivated for 24 h. After cell attachment, plates were washed with PBS, and incubated with H_2_O_2_ (500 µM) for 2 h prior to treatment with increasing concentrations of AA extract prepared in 10% FBS containing media for 12 h. The stimulated cells were washed with PBS and incubated for 30 min with dichlorfluorescein diacetate (DCFDA) dissolved in DMSO (final concentration 50 µM). Fluorescence was measured at 0 and 40 min using an excitation of 485 nm and emission of 535 nm, in fluorescence microplate reader (Infinite 200, Tecan, Grodig, Austria). Most of steps including incubation of reaction mixture containing dye and oxidant, washing and fluorimetric determination were performed in the dark. The intensity of fluorescence was calculated as [(F40 min–F0 min)/F0 min] ×100 as described elsewhere [18]. Results are expressed as relative intensity of fluorescence (in % of negative control).

### Measurements of serum oxidative markers and antioxidant potential

The level of malondialdehyde (MDA) in liver tissue homogenates was determined using assay kit (OxiSelectTM TBARS assay kit, San Diego, CA, USA) based on the method of Mihara and Uchiyama [19]. Briefly, Half a milliliter of each tissue homogenate was mixed with 3 ml of H_3_PO_4_ solution (1%, v/v) followed by addition of 1 ml of thiobarbituric acid solution (0.67%, w/v). The mixture was incubated at 95°C in a water bath for 45 min. The colored complex was extracted into n-butanol, and the absorption at 532 nm was measured using tetramethoxypropane as standard. MDA levels were expressed as nmol per milligram of protein.

The serum level of 8-OHdG level, a biomarker of oxidative DNA damage, was assessed by 8-OH-dG ELISA kit (Assay Designs/Stressgen, Ann Arbor, MI, USA).

Ferric reducing potential of plasma (FRAP) of the sample was measured as redox-linked colorimetric method [20]. In this assay, a ferric-2,4,6-tripyridyl-s-triazine (Fe III-TPTZ) complex reduced to the ferrous ion, which is blue colored. 30 µL of FRAP working reagent containing 300 mM of acetate buffer (pH 3.6), 10 mM Fe III-TPTZ in 40 mM HCl and 20 mM FeCl_3_•6H_2_O in the ratio of 10∶1∶1 was mixed with 7 µL of sample or standard and was added 170 µL of distilled water in a 96-well microplate. After incubation for 10 min at room temperature, the absorbance at 593 nm was detected by ELIZA reader (Tecan Sunrise microplate reader, ReTiSoft Inc., Mississauga, Ontario, Canada). α-Tocopherol and trolox were used as a positive control.

Total phenolics were determined using Folin-Ciocalteu reagent [21]. Briefly, 30 mg of sample was extracted for 18 h with 50 mL of aqueous ethanol at room temperature on an orbital shaker set at 200×g. The mixture was filtered through Whatman filter paper and used for total phenolics assay at a total volume of 50 mL. Four hundred microliters of extract was mixed with 50 µL of Folin-Ciocalteu reagent (previously diluted 5-fold with distilled water) and 100 µL of saturated sodium bicarbonate solution and then allowed to stand at room temperature for 1 h. The absorbance was measured at 750 nm. Results are expressed as gallic acid equivalents (GAE).

### Assessment of NQO1 activity

Mouse hepatoma hepa1c1c7 cells were treated with various concentrations of AA extract for 24 h and subjected to the measurement of antioxidant enzyme NQO1 activity.

The enzyme activities of mouse tissues and cell homogenates were measured by a spectrophotometric assay in which the rate of reduction of 2,6-dichlorophenolindophenol was monitored at 600 nm [22]. The specific activity of enzymes was normalized to the protein concentration, which was determined in duplicate using the Lowry method [23]. All values were expressed as mean ± standard deviation (SD).

### Western blotting

After 30 min of incubation on ice, the tissue homogenates were cleared by centrifugation at 14,000 g for 10 min at 4°C, and the supernatants were denatured in sample buffer for 5 min at 95°C. Proteins were separated by electrophoresis on a 10% SDS-polyacrylamide gel for 1.5 h at 100 V and transferred onto nitrocellulose membranes (Amersham Biosciences, Freiburg, Germany) for 1 h at 100 V. Membranes were incubated with antibodies to anti-NQO1, anti-β-actin at dilutions of 1∶1,000 overnight at 4°C. The bands were detected using a chemiluminescence kit (Pierce, Cheshire, United Kingdom). Densitometry analysis was performed with Lab Image software.

### Statistical analysis

Statistical significance of data was tested by analysis of variance, followed by Duncan' multiple range test, using SPSS software (SPSS Inc., Chicago, IL). Data were presented as mean ± standard error (SE). Means sharing common letter represent no statistical significance, *P*<0.05.

## Results

### Artemisinin content and antioxidative activity of *A. anua* extract

When AA powder was extracted with 10 volumes of aqueous ethanol solution with different ethanol concentrations, the artemisinin content in the extract was increased in proportion to ethanol concentration in the solvent, with 0.50 mg/g dry matter in 80% (v/v) ethanol extract.

Meanwhile, total phenolic content of AA extract was the highest when 80% (v/v) ethanol solution was used as a solvent among various aqueous ethanol solutions, with 32.67±2.84 mg (mean ± SD) gallic acid equivalent per gram dry matter.

**Figure 1:**
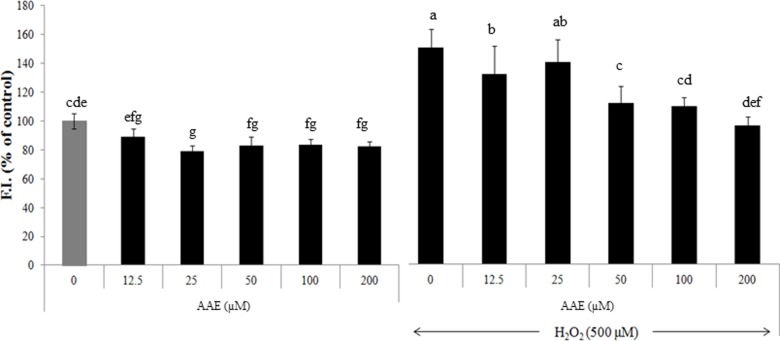
Reduction of intracellular ROS level by AA extract. Human hepatoma HepG2 cells were exposed to either AA extract or AA extract plus hydrogen peroxide (500 µM), followed by measuring fluorescence generated by adding DCFDA. Values are means ± SD (n = 10). Means without a common letter differ, *P*<0.05.

Pre-incubation of HepG2 cells in the presence of AA extract for 12 h reduced hydrogen peroxide-induced ROS production in a dose-dependent manner as assessed by DCFDA method in which intracellular ROS generates fluorescence upon reaction with DCF (Fig. 1). AA extract itself did neither show any cytotoxicity nor affected intracellular ROS level in HepG2 cells grown in the absence of hydrogen peroxide even at the maximum concentration of 200 µg/mL used in the study.

### Induction of NQO1 activity by *A. annua* extract

**Figure 2:**
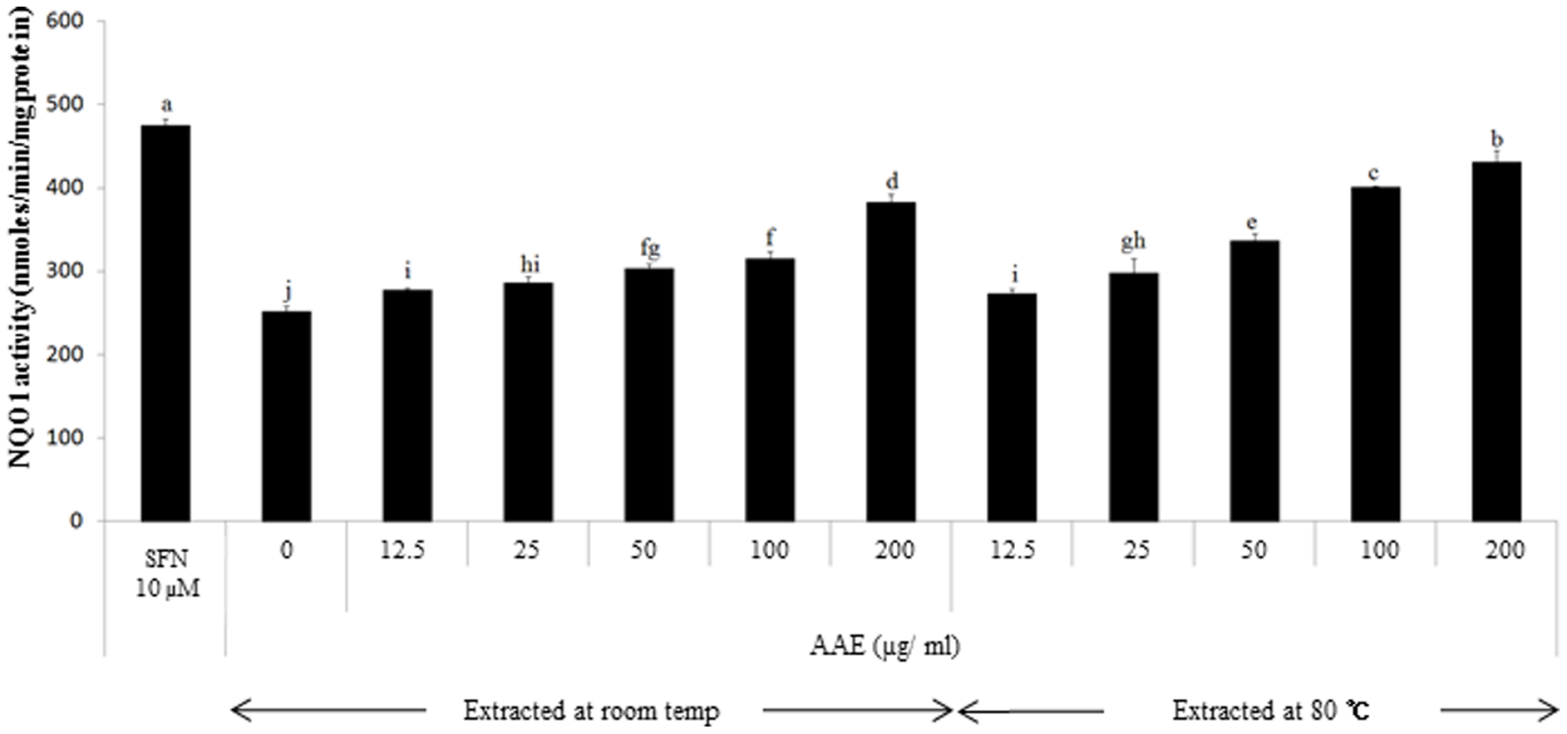
Induction of NQO1 activity by AA extract in hepa1c1c7 cells. Mouse hepatoma 1c1c7 cells were exposed to 10 µM sulforaphane (SFN) as a positive control and different doses of AA extract for 24 h, and subjected to NQO1 assay as described in Materials and Methods. Values are means ± SD (n = 10). Means without a common letter differ, *P*<0.05.

The enzyme activity of NQO1, one of the antioxidant and anticarcinogenic biomarker enzymes, was dose-dependently induced by AA extract in the range of 12.5 to 200 µg/mL in murine hepatoma hepa1c1c7 cells (Fig. 2). The NQO1 activity was increased by ∼20% and ∼60% in hepa1c1c7 cells treated with the concentrations of 25 and 200 µg/mL of AA extract at both extraction temperatures of room and 80°C, respectively, while the enzyme activity was enhanced by 90% by 10 µM sulforaphane, a well-known NQO1 inducer.

### Effect of AA extract-containing diet on oxidative damage markers in mouse

**Figure 3:**
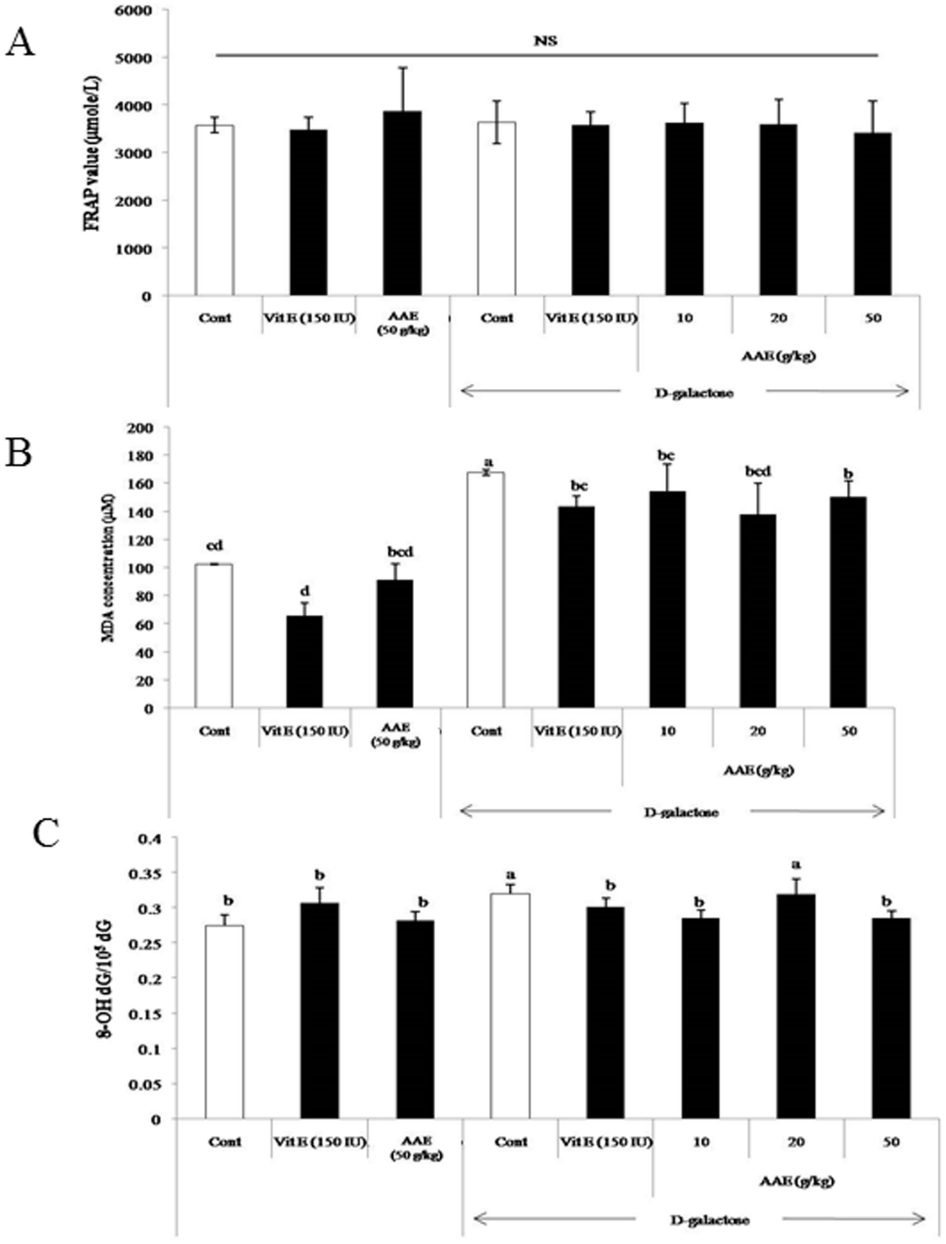
Effect of AA extract diet on oxidative biomarkers FRAP, MDA and 8-OH-dG levels in serum from mice chronically exposed to D-galactose. C57BL/6J mice (5-week old, n = 10) were treated with AA extract with and without D-galactose exposure for 6 weeks, and then oxidative biomarkers including serum MDA and 8-OH-dG levels were measured according to the protocol provided by the suppliers of assay kits. Values are means ± SE (n = 10). Means without a common letter differ, *P*<0.05.

None of the mice tested had obvious health problems including weight loss, cataracts, or toxicity reaction. FRAP was not significantly different among the experimental groups (Fig. 3A), regardless of the treatment of D-galactose or AA extract.

The serum MDA level, a biomarker for lipid peroxidation, in D-galactose-treated mice was significantly increased and reduced by co-treatment with AA extract as shown in Fig. 3B.

The plasma level of 8-OH-dG, a biomarker for DNA damage, was also increased in mice treated with D-galactose and reduced in the groups fed diets containing AA extract. However, the decreased serum level in MDA and 8-OH-dG in AA extract-fed groups was not dose-dependent, without a significant difference between the groups fed diet containing 10 or 50 mg AA extract per kg diet.

### Induction of NQO1 activity by *A. annua* extract in mice

**Figure 4:**
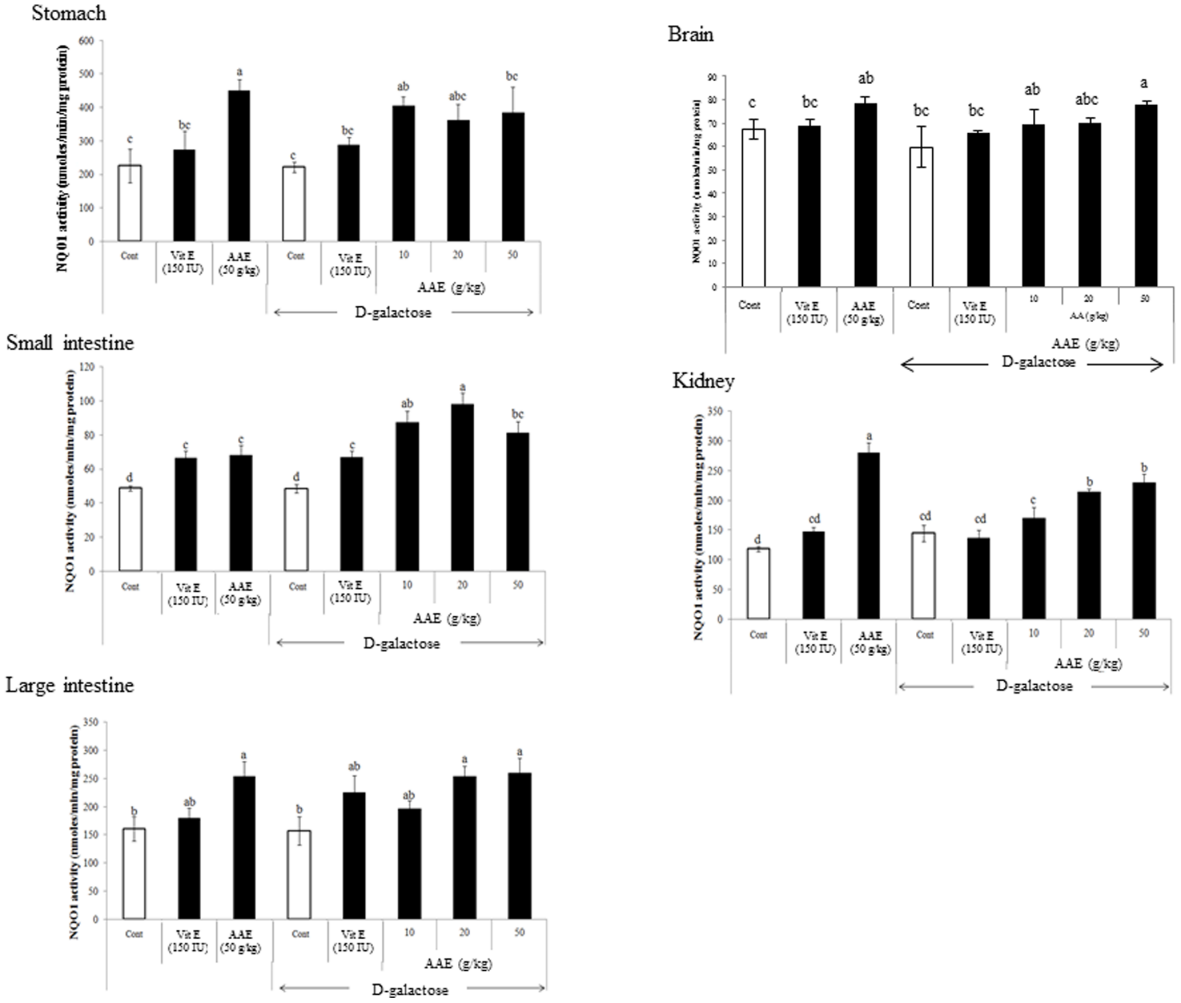
Effect of AA extract diet on NQO1 activity in mouse tissues. C57BL/6J mice (5-week old, n = 10) were treated with AA extract with and without D-galactose exposure for 6 weeks, and then subjected to assay for NQO1 enzyme activity in stomach, small intestine, large intestine, kidney, and brain. Values are means ± SE (n = 10). Means without a common letter differ, *P*<0.05.

**Figure 5:**
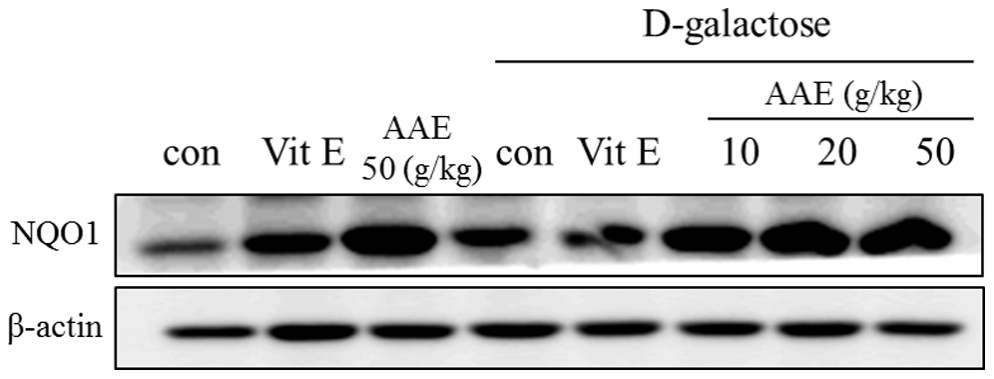
Increased NQO1 expression in small intestine of mice administered with AA. C57BL/6J mice (5-week old, n = 10) were treated with AA extract with and without D-galactose exposure for 6 weeks, and then epithelium of small intestine was scraped and subjected to western blot for the determination of NQO1 level.

The NQO1 activity in organs from mice fed diet containing AA extract was increased significantly (Fig. 4). Induction of the enzyme activity was most prominent in kidney tissue and small intestinal mucosa. While D-galactose itself did not affect NQO1 activity, AA extract dose-dependently induced the enzyme activity, in particular, in small intestine from mice treated with D-galactose. The NQO1 protein expression in small intestine was also significantly enhanced by AA treatment (Fig. 5), suggesting that the enhancement of NQO enzyme activity by AA extract was mediated by increased protein expression.

## Discussion

*A. annua* L. is well-known to contain artemisinin and its derivatives which are a family of sesquiterpene trioxane lactone anti-malarial agents [3]. The herb is also popular as a source of essential oils which mainly consist of camphor (44%), germacrene D (16%), trans-pinocarveol (11%), β-selinene (9%), β-caryophyllene (9%) and Artemisia ketone (3%). The essential oil remarkably inhibited the growth of tested Gram-positive bacteria *Enterococcus hirae*, and fungi *Saccharomyces cerevisiae* and *Candida albicans* [24]. In addition, the oils from Artemisia genus have shown a moderate antioxidant activity [24], [25].

It has been reported that *A. annua* contained a significant level of phenolic compounds including luteolin, luteolin-7-glucoside, kaempferol, quercetin, rutin, coumarin and so on [26]. The present study also showed that the ethanol extract of the herb contained 32.67±2.84 mg total phenolics per g dry matter, consistent with the previous report that the herb had 1.54 mg total phenolics per g fresh weight [27].

The present study confirmed antioxidant activity of 80% (v/v) ethanol extract of the herb in cultured cell and mouse model systems. The serum level of 8-OH-dG increased by D-galactose injection was restored to the untreated control level by feeding diet containing AA extract. The D-galactose exposure has been reported to induce an increase in peripheral oxidative stress, including an increase in malondialdehyde (MDA) and decreases in total antioxidative capabilities, total superoxide dismutase, and glutathione peroxidase activities [28]. Our study also confirmed that the MDA and 8-OH-dG levels were significantly enhanced by D-galactose in mouse and were decreased upon treatment with either α-tocopherol or AA extract. A chronic administration with a low dose of D-galactose is reported to induce changes that mimics natural aging in animals, such as a shortened life span [29], [30], cognitive dysfunction [31], neurodegeneration [32], and oxidative stress [28], [33]. The protective effect of AA extract from lipid peroxidation and DNA damage appears to be associated with the capability of AA extract to induce antioxidant enzymes including NQO1. That is, it was well established that antioxidant enzymes were induced by some phytochemicals in an Nrf2-mediated fashion. More specifically, some electrophiles including sesquitepenes interact with Keap1 that is present in heterodimeric form with Nrf2 in cytosol, releasing Nrf2 from the complex. The released Nrf2 migrates into the nucleus and act as a transcriptional factor, promoting expression of antioxidant enzymes such as NQO1, heme oxygenase 1, glutathione reductase, γ-glutamyl cysteine ligase, and glutathione *S*-transferase and so on [34]–[36]. The current study also demonstrated that AA extract increased the NQO1 activity and expression in mouse organs such as stomach, small intestine, and large intestine, and kidney. Though the activities of the other antioxidant enzymes were not measured, it is most likely that the herb extract had ability to induce antioxidant enzymes in Nrf2-mediated fashion in the tissues as NQO1 is one of the typical antioxidant enzymes downstream to Nrf2 signaling pathway. While the constituents responsible for NQO1 induction in mouse tissues remain unclear, there is possibility that artemisinin and its derivatives played a role in the enzyme induction because artemisinin content in the sample was 1.6 mg/g that is enough to affect Nrf2 signaling [37].

In addition, NQO1 antioxidant enzyme in tissues from mice fed *Artemisia annua* extract may be attributable to phenolic compounds such as flavonoids and coumarins that are electrophiles with antioxidant activity and hold capability to induce phase 2/antioxidant enzymes through activating Nrf2 signaling pathway [38].

In conclusion, AA extract showed antioxidative activity in hepatoma cells as well as protected from lipid peroxidation and DNA damage in D-galactose-induced oxidative stress mouse model. Therefore, it deserves further clinical study to be developed into health functional ingredient.
